# A Critical Assessment of Marine Aquarist Biodiversity Data and Commercial Aquaculture: Identifying Gaps in Culture Initiatives to Inform Local Fisheries Managers

**DOI:** 10.1371/journal.pone.0105982

**Published:** 2014-09-10

**Authors:** Joanna M. Murray, Gordon J. Watson

**Affiliations:** 1 Environment and Ecosystems, Centre for Environment, Fisheries and Aquaculture Science, Lowestoft, Suffolk, United Kingdom; 2 Institute of Marine Sciences, School of Biological Sciences, Portsmouth, Hampshire, United Kingdom; The Australian National University, Australia

## Abstract

It is widely accepted that if well managed, the marine aquarium trade could provide socio-economic stability to local communities while incentivising the maintenance of coral reefs. However, the trade has also been implicated as having potentially widespread environmental impacts that has in part driven developments in aquaculture to relieve wild collection pressures. This study investigates the biodiversity in hobbyist aquaria (using an online survey) and those species currently available from an aquaculture source (commercial data and hobbyist initiatives) in the context of a traffic light system to highlight gaps in aquaculture effort and identify groups that require fisheries assessments. Two hundred and sixty nine species including clown fish, damsels, dotty backs, angelfish, gobies, sea horses and blennies, have reported breeding successes by hobbyists, a pattern mirrored by the European and US commercial organisations. However, there is a mismatch (high demand and low/non-existent aquaculture) for a number of groups including tangs, starfish, anemones and hermit crabs, which we recommend are priority candidates for local stock assessments. Hobbyist perception towards the concept of a sustainable aquarium trade is also explored with results demonstrating that only 40% of respondents were in agreement with industry and scientists who believe the trade could be an exemplar of a sustainable use of coral reefs. We believe that a more transparent evidence base, including the publication of the species collected and cultured, will go some way to align the concept of a sustainable trade across industry stakeholders and better inform the hobbyist when purchasing their aquaria stock. We conclude by proposing that a certification scheme established with government support is the most effective way to move towards a self-regulating industry. It would prevent industry “greenwashing” from multiple certification schemes, alleviate conservation concerns, and, ultimately, support aquaculture initiatives alongside well managed ornamental fisheries.

## Introduction

Coral reefs are increasingly suffering from a plethora of impacts including rising sea temperatures, over fishing, sedimentation and pollution [1]. The marine aquarium trade has been implicated as having potentially widespread and long-lasting environmental and biological impacts; so it is no surprise that growth of the industry is perceived as a direct threat to their conservation [2], [3]. With reports that a number of species have seen population declines in response to their collection [3], [4], [5], [6], [7] the over exploitation of target populations is of major concern to conservationists, and exacerbated by the absence of monitoring or regulatory control. However, the trade is highly species specific and low volume and therefore has great potential to provide livelihoods to coastal communities whilst incentivising maintenance of a healthy coral reef [8], [9]. To truly champion the aquarium trade as a sustainable industry with important socio-economic benefits, a requirement remains for development of a robust scientific evidence base to underpin its management through an ecosystem-based approach.

Trade in marine ornamental species has grown dramatically during the last decade with an estimated 10 million invertebrates (excluding corals) collected every year [2], [3], [10]. Moreover, the actual extent of the industry is likely to be much larger with a recent study by Murray et al. [11] suggesting that this number may be as much as a 10–20 fold underestimate. The increasing demand for ornamental invertebrates has largely been driven by a shift in consumer preferences over the last 10–15 years from a fish-only system to miniature reefs [3], [12]. Technological advances especially in lighting have led to a reduction in the size of viable aquariums using these systems (e.g. pico and nano aquariums), lowering the cost of aquaria and consequently increasing demand [13]. To supply an aesthetically pleasing and ecologically functioning mini-reef ecosystem, ornamental fishermen (collectors) supplying the trade are utilising the full suite of coral reef biodiversity and have been described by Rhyne et al. [3], as “a generalist predator that targets both abundant and rare species with a premium on biodiversity and scarcity *per se*”. Although there have been some recent studies to assess trade volume for some ornamental species (e.g. [3], [11]), these have focussed on the collection and supply chain sectors of the industry. An evaluation of species diversity at the final point in the chain of custody (i.e. within hobbyists' aquaria) is still lacking. The first part of this study addresses this issue using an online survey of hobbyists to assess the biodiversity for major groups of both fish and invertebrates held in aquaria and the motivations for making particular consumer choices.

Growing concern over the sustainability of the aquarium industry has prompted developments in aquaculture as an alternative to wild collection for meeting market demand [14], [15], [16]. Recent reviews [17], [18] have focussed on the development of culture techniques and processes but have not directly assessed the current capacity of the aquaculture industry to supply cultured alternatives. Data gathered from online hobbyist-driven sources (e.g. the Marine Breeders Initiative) and a number of commercial organisations have enabled us to produce an assessment of aquaculture potential for key families and groups, as well as identify those groups which are harvested in low numbers (low concern) and, and those which may require fisheries management.

The final part of this study assesses hobbyist attitudes towards the concept of a sustainable aquarium trade as well as their support for aquaculture and willingness to pay a ‘green’ price premium for cultured organisms. Combined with the industry data this information enables us to assess if hobbyist preferences and expectations for cultured products match what is currently provided (e.g. the cultured organisms available) by the industry as well as highlight candidates of ornamental species for local fisheries assessments.

## Materials and Methods

### Study design and subjects

Survey data were collected through an online questionnaire which was available from April 29^th^ 2009. Survey design software (Surveymonkey.com) was used to create a 21-question survey in a variety of formats including; multiple choice with restricted or multiple answer options, rating scales and text boxes. Questions were divided into four sections: respondent demographics; the hobbyist aquarium; purchasing preferences and the concept of “sustainability”; and the future (see Table S1 for all questions). A personalised link taking the respondent directly to the questionnaire was generated and advertised on Practical Fishkeeping Magazine's official website for five days and printed in their June 2009 issue of the magazine. The link was also posted on ten marine fish keeping fora which were selected based on the top fora defined from an internet search for “marine fish keeping forum”. These included: Nanoreefs; Saltwater aquariums; ReefFace; Marine fish forum; Ultimate reef; Aquaria central; Tropical fish forums; Total fishkeeping; International reefers; and Fishing keeping forum. The online survey was left active for a period of 4 months before the raw responses were downloaded.

### Questionnaire

General questions to obtain the demographics of the responding hobbyist including: the sex, age and country of residence were asked. These were followed by a series of multiple-choice restricted and text-box questions aimed at assessing the hobbyists' aquarium including: their personal reasons for keeping an aquarium and the geographical region/species on which it is based; the most important factors when buying a new aquarium animal using a rating scale; preferences for a “sustainable” aquarium trade and the perceived view of the future of the industry. Key questions asked in the questionnaire can be found in Table S1.

### Commercial data and online databases

Information on the commercial culture of species was gathered (primarily by email) from companies within the European and USA trade and using scientific literature (Web of Knowledge) for relevant research papers on ornamental aquaculture. FishBase and the World Register of Marine Species were used to assign the taxonomic classes [19], [20]. The Marine Breeders Initiative (MBI) (a project of the Marinelife Aquarium Society of Michigan) was created as a tool to encourage marine aquaria hobbyists to get involved in the captive breeding of marine organisms and document their successes. Data from the list were used directly (with guidance from T. Sweet, Marinelife Aquarium Society of Michigan) to ascertain the number of captive breeding reports. At the time of access (April 2013) 391 species were recorded on the database. To meet the requirement of ‘successful’, members (hobbyists) submit reports on categories including: ‘spawning’; ‘hatching’; ‘larval settlement’; and ‘60 days post-larval settlement’. Records of all categories were included as successful for this study but all records of asexual reproduction (e.g. fragging, budding and fission etc) were not recorded.

### Hobbyist biodiversity data and aquaculture potential

The percentage of hobbyists stocking key groups/families of marine ornamental fish and invertebrates was calculated from data submitted through the online survey. The potential for producing these groups using aquaculture was then assessed based on information from commercial companies and knowledge of the species life histories. Groups containing species that are commercially cultured at present (e.g. clown fish, damsels, angel fish etc) were assessed as having a high potential for culture. Those groups with a medium score have a small number (one or two species currently cultured, e.g. jaw fish, look downs, trevally, skillet fish, cling fish and sea urchins), but have potential for transfer to similar species within the group. Fan worms were included in this level based on current pilot-level culture implementation using sexual reproduction [21], [22], [23], [24] and regeneration [16]. The remaining groups were classified as having low aquaculture potential as no commercial organisations have reported successful reproduction and very few/no reports of breeding were recorded on the MBI list.

### Aquaculture gap analysis

Gap analysis of the current culture status of the family/group's kept by the hobbyist was performed using data on the demand for each ornamental group (the number of fish species imported into the USA during one year (2004-5) modified from Rhyne et al. [25], and hobbyist aquaria biodiversity data obtained from the current study) in combination with data on the number of species currently cultured using data on the number of species cultured by commercial companies and records of culture successes from the Marine Breeders Initiative (MBI). Expert judgement was used to assess this evidence and assign each group/family to a traffic light system (green, amber or red category) based on hobbyist demand and current operational efforts to culture them. A green category included species with low demand or medium/high demand but successful aquaculture production; an amber category included those species with medium or high demand and limited aquaculture potential, while a red category highlights high demand groups with no operational culture initiatives.

### Statistical analyses

Data were analysed using Minitab and SPSS V. 15. Mean rank were analysed with non-parametric methods (Kruskal Wallis with multiple comparisons) to detect differences in consumer beliefs. All data were presented using SigmaPlot 2000.

## Results

### Respondent demographics

Three hundred and fourteen people responded to the online questionnaire, 68 of which were women (21.7%) and 246 were men (78.3%) (Table 1). Sixty four percent were between the ages of 21 and 40 years old. Although the survey was available worldwide, 77% of respondents were from the UK, 13% from the USA and the remainder were from ten other countries. Online forums accounted for the largest percentage of completed questionnaires with 61% of the survey population originating from an online forum link and 33% of the remaining respondents identified the Practical Fishkeeping magazine website as their information source. When combined with online fora, 94% of all respondents had encountered the questionnaire on the internet.

**Table 1:** Survey sample demographics.

Gender	
Male	78.3
Female	21.7
Age (years)	
≤20	12.1
21–40	62.7
40–60	22.6
60+	1.9
Un-answered	0.7
Country of residence	
United Kingdom	77.1
United States of America	13.4
Canada	2.5
Australia	1.6
India	1
Ireland	1
Sweden	1
Other	1.6
Un-answered	1
Where did you find the questionnaire	
Advert in hobbyist magazine	32.8
Online hobbyist site	3.8
Online forum	60.8
Other	2.2
Un-answered	0.4

Demographic information about the online survey respondents (%, n = 314) regarding: gender; age; country of residence; position in the trade; and how they encountered the survey

### What's in hobbyist aquaria?

Three hundred of the respondents owned a reef-based aquarium and only 27 a fish-only system. Ten percent of those owning a reef aquarium had used a specific region, the most popular of which included the Caribbean, the Great Barrier Reef and the Indo-Pacific. Clown fish (Pomacentridae) were the most popular fish with an 84% response rate and between 32% and 53% of respondents stated ownership of gobies (Gobiidae), angelfish (Pomacanthidae), blennies (Blenniidae), damsel fish (Pomacentridae) and wrasse (Labridae) (Table 2). Fewer than 10% of all respondents recorded butterfly fish (Chaetodontidae), groupers (Serranidae), seahorses (Syngnathidae) and trigger fish (Ballistidae) as aquarium inhabitants. Other fish species such as dragonets (Callionymidae), lion fish (Scorpaenidae) and hawk fish (Cirrhitidae) were listed in the ‘others’ category but have been separated for the table.

**Table 2:** Biodiversity and aquaculture potential of fish in the marine aquarium trade.

Organism group	% hobbyist	Family or appropriate taxonomic class	Species imported to USA	Stock demand	Captive bred reports	Organisation									Asex repro.	Future AC potential	Gap Analysis
						1	2	3	4	5	6	7	8	9			
Clown fish	85	Pomacentridae	170	H	25 (32%)	1	5	3	15	0	19	8	4	12	-	H	Green
Damsels	34			H	29 (32%)	19	23	0	2	2	1	0	0	1	-	H	
Tangs	57	Acanthuridae	57	H	0	0	0	0	0	0	0	0	0	0	-	L	**Red**
Gobies	52	Gobiidae	138	H	27 (20%)	5	25	0	2	0	13	6	0	0	-	H	Green
Wrasse	44	Labridae	228	H	1 (0.43%)	0	0	0	0	0	0	0	0	0	-	L	**Red**
Basselets		Grammatidae	4	H	2 (50%)	0	2	0	0	0	0	3	0	0	-	H	Green
Dotty backs		Pseudochromidae	37	H	24 (65%)	5	16	0	9	0	18	5	0	0	-	H	Green
Grammas		Serranidae	131^a^	H	6 (4.5%)	0	0	0	0	0	0	0	0	0	-	L	**Red**
Blennies	37	Blenniidae	77	H	17 (22%)	7	10	0	5	20	8	0	0	0	-	H	Green
Angel fish	33	Pomacanthidae	66	H	21 (32%)	0	6	0	3	2	0	0	0	1	-	H	Green
Cardinal fish	23	Apogonidae	66	M	8 (12%)	2	4	1	2	0	6	2	1	1	-	H	Green
Trigger fish	9	Balistidae	27	M	2 (7%)	0	0	0	0	0	0	0	0	0	-	L	*Amber*
Butterfly fish	9	Chaetodontidae	97	M	0	0	0	0	0	0	0	0	0	0	-	L	*Amber*
Puffer fish, box fish	5	Tetraodontidae	31	M	3 (10%)	0	0	0	0	0	0	0	0	0	-	L	*Amber*
Sea horses, pipe fish	5	Syngnathidae	23	M	23 (100%)	2	11	0	5	0	9	2	1	0	-	H	Green
Groupers, sand fish	2	Serranidae	131^a^	L	4 (3%)	0	0	0	1	0	0	0	0	0	-	L	Green
Dragonets, mandarins	1.3	Callionymidae	16	L	6 (38%)	0	2	0	0	0	4	3	0	0	-	H	Green
Assesors, bettas	1	Plesiopidae	12	L	4 (33%)	1	4	0	1	0	5	1	0	0	-	H	Green
Worm fish	0.3	Microdesmidae	12	L	0	0	0	0	0	0	0	0	0	0	-	L	Green
Lion fish	0.3	Scorpaenidae	35	L	0	0	0	0	0	0	0	0	0	0	-	L	Green
Hawk fish	0.3	Cirrhitidae	15	L	0	0	0	0	0	0	0	0	0	0	-	L	Green
File fish	0.3	Monacanthidae	26	L	2 (8%)	0	0	0	0	0	0	0	0	0	-	L	Green
Eels	0.3	Muraenidae	47	L	0 (0%)	0	0	0	0	0	0	0	0	0	-	L	Green
Rabbit fish	0	Siganidae	19	L	5 (26%)	0	0	0	0	0	0	0	0	0	-	L	Green
Grunts	0	Haemulidae	29	L	2 (7%)	0	0	0	0	5	1	2	0	0	-	H	Green
Catfish	0	Plotosidae	3	L	1 (33%)	0	0	0	0	0	0	0	0	0	-	L	Green
Sharks, rays	0	Hemiscylliidae, Dasyatidae, Heterodontidae, Scyliorhinidae	21	L	8 (38%)	1	0	0	2	0	0	0	0	0	Y^f^	H	Green
Razor fish	0	Centriscidae	2	L	1 (50%)	0	0	0	0	0	0	0	0	0	-	L	Green
Toad fish, frog fish	0	Batrachoididae	4	L	1 (25%)	0	0	0	0	0	0	0	0	0	-	L	Green
Snappers	0	Lutjanidae	30	L	1 (3%)	0	0	0	0	0	0	0	0	0	-	L	Green
Jaw fish	0	Opistognathidae	4	L	1 (25%)	0	1	0	0	0	0	1	0	0	-	M	Green
Look down, trevally	0	Carangidae	14	L	2 (14%)	0	2	0	1	0	0	0	0	0	-	M	Green
Skillet fish, cling fish	0	Gobiesocidae	4	L	2 (50%)	1	0	0	0	0	0	0	0	0	-	M	Green
Dart fish, fire fish	0	Ptereleotridae	2	L	1 (50%)	0	0	0	0	0	0	0	0	0	-	L	Green
Cow fish	0	Ostraciidae	16	L	1 (6%)	0	0	0	0	0	0	0	0	0	-	L	Green
Bat fish, spade fish	0	Ephippidae	5	L	3 (60%)	0	2	0	2	2	0	1	0	1	-	H	Green
Drum, jack-knife fish	0	Sciaenidae	4	L	4 (100%)	0	0	0	0	0	0	0	0	0	-	L	Green
Moon fish	0	Monodactylidae	1	L	0 (0%)	0	0	0	0	0	0	0	0	0	-	L	Green

*Organism group*: labels from online survey groupings. *Percentage hobbyist*: number of hobbyists from online survey that keep specimens of that group. *Species imported to USA*: number of species imported to USA for 2004/5 using data from Rhyne et al. [25] (^a^groupers and sandfish are also part of Serranidae but Rhyne et al. [25] did not distinguish between the two). *Stock demand*: defined as percentage of organism group kept by hobbyist with low (<2%); medium (2–25%) and high (>25%). *Captive bred reports*: reports of successful sexual reproduction from the Marine Breeders Initiative list (accessed April 2013) and the percentage of species imported into the USA. *Organisations 1-9*: who provided information on species cultured commercially (not all are currently in commercial production). *Other sources*: other records (e.g. research articles etc) of reproduction. *Asexual reproduction*: records of species that can reproduce asexually (^f^shark parthenogenesis e.g. [19]). *Aquaculture potential*. Low: no commercial breeding, very few or no reports of breeding on MBI list. Aquaculture is not likely to fill the gaps any time soon. Medium: some potential, a few species bred but minimal commercial output; High: many species already bred on MBI list and commercially common, expect rapid transfer to other species in family assuming similar breeding pattern. *Gap Analysis*. Traffic light system (green, amber or red category) based on hobbyist demand and current operational efforts to culture them. Green category includes species with low demand or medium/high demand but successful aquaculture production; amber category included those species with medium or high demand and limited aquaculture potential, red category highlights high demand groups with no operational culture initiatives.

The number of respondents stocking an invertebrate species (Table 3) was higher than those stocking many fish species (excluding clown fish), with over 70% of hobbyists owning soft (Alcyonacea, Corallimorpharia, Zoantharia, Octocorallia) and hard corals (Scleractinia, Antipatharia), crabs (Brachyura), snails (Gastropoda) and shrimp (Hippolytidae, Hymenoceridae). Snails were the most popular invertebrate choice with 85% of all respondents keeping them. Starfish (Asteriodea), anemones (Actiniaria, Ceriantharia), giant clams (Tridacninae) and fan worms (Sabellidae) were less popular but were still present in over 25% of respondent's aquariums.

**Table 3:** Biodiversity and aquaculture potential of invertebrates in the marine aquarium trade.

Organism group from online survey	% hobbyist	Family or appropriate taxonomic class	Species imported to USA	Stock demand	Captive bred reports	Organisation									Other sources	Asex repro.	Future AC potential	Gap Analysis
						1	2	3	4	5	6	7	8	9				
Soft corals	86	Alcyonacea, Corallimorpharia, Zoantharia, Octocorallia	-	H	3	0	0	24	17+	0	0	0	8	3	Hobby	Y^g^	H	Green
Snails	85	Gastropoda	-	H	5	0	0	0	0	0	3	0	0	0	Y^b, c^	-	H	Green
Crabs, hermit crabs	79	Decapoda	-	H	1	0	0	0	0	0	0	0	0	0	Y^d^	-	L	**Red**
Shrimp	76	Hippolytidae, Hymenoceridae	-	H	9	1	4	0	3	0	2	0	2	0	-	-	H	Green
Hard corals	71	Scleractinia, Antipatharia	-	H	0	0	0	132	28+	0	0	0	0	83	Hobby	Y^g^	H	Green
Starfish	53	Asteroidea	-	H	6	0	0	0	0	0	0	0	0	0	-	-	L	**Red**
Feather dusters	44	Sabellidae	-	H	0	0	0	0	0	0	0	0	0	0	Y^e^	Y^h^	M	*Amber*
Anemones	39	Actiniaria, Ceriantharia	-	H	1	0	0	0	0	0	0	0	0	0	-	Some^i^	M	**Red**
Clams	25	Tridacninae	-	H	0	0	0	0	5	0	6	0	1	0	-	-	H	Green
Koko worms	8	Serpulidae	-	M	0	0	0	0	0	0	0	0	0	0	-	-	L	*Amber*
Sea urchin	1.3	Echinoidea	-	L	0	0	0	0	0	0	1	0	0	0	-	-	M	Green
Other mollusc (nudibranch)	1.3	Nudibranchia	-	L	4	1	0	0	0	0	0	0	0	0	-	-	M	Green
Other polychaete	1	Polychaeta	-	L	0	0	0	0	0	0	0	0	0	0	-	-	L	Green
Mantis shrimp	0.6	Stomatopoda	-	L	0	0	0	0	0	0	0	0	0	0	-	-	L	Green
Sponge, tunicate	0.3	Porifera, Tunicata	-	L	0	0	0	0	0	0	0	0	0	0	-	-	L	Green
Other crustacean	0.3	Decapoda	-	L	0	0	0	0	0	0	0	0	0	0	-	-	L	Green

*Organism group*: labels from online survey groupings. *Percentage hobbyist*: number of hobbyists from online survey that keep specimens of that group. *Species imported to USA*: number of species imported to USA for 2004/5 using data from Rhyne et al. [25]. *Stock demand*: defined as percentage of organism group kept by hobbyist with low (<2%); medium (2–25%) and high (>25%). *Captive bred reports*: reports of successful sexual reproduction from the Marine Breeders Initiative list (accessed April 2013) and the percentage of species imported into the USA. *Organisations 1-9*: who provided information on species cultured commercially; not all are currently in commercial production. *Other sources*: other records (e.g. research articles etc) of reproduction (^b^gastropods e.g. [30], [31]; ^c^using non-tropical species e.g. [34]; ^d^ *Mithraculus* crabs e.g. [24], [25], [26]; ^e^ *Sabellastarte spectabilis* e.g. [21], [22], [23], [24]. *Asexual reproduction*: records of species that can reproduce asexually ^g^ produced by fragging; ^h^ by regeneration e.g. [16]; ^i^some anemones e.g. [27], [28]. *Aquaculture potential*. Low: no commercial breeding, very few or no reports of breeding on MBI list. Aquaculture is not likely to fill the gaps any time soon. Medium: some potential, a few species bred but minimal commercial output; High: many species already bred on MBI list and commercially common, expect rapid transfer to other species in family assuming similar breeding pattern. *Gap Analysis*. Traffic light system (green, amber or red category) based on hobbyist demand and current operational efforts to culture them. Green category included species with low demand or medium/high demand but successful aquaculture production; amber category included those species with medium or high demand and limited aquaculture potential, while a red category highlights high demand groups with no operational culture initiatives.

### Species availability from a cultured source

Two hundred and sixty nine species have entries for breeding success on the MBI list, the vast majority of which are fish. Seven groups, clown fish, damsels, dotty backs, angelfish, gobies, sea horses and blennies, had over 15 records from different species exhibiting high potential for aquaculture Other groups with a high potential for the future aquaculture were the dragonets and mandarins, cardinal fish (Apogonidae), puffer and box fish (Tetraodontidae) and rabbit fish (Siganidae). Key species which have no breeding success recorded in the database and low aquaculture potential include the tangs (Acanthuridae), butterfly fish, lion fish, worm fish (Microdesmidae) and hawk fish. Only 29 invertebrate culture successes were recorded on the MBI list, with shrimps, snails and starfish having the most reports. Groups with notably few or absent reports include the sea urchins (Echinoidea), crabs and hermit crabs, all polychaetes (including the Sabellidae and Serpulidae) and hard corals.

Nine organisations, five from the USA and four from Europe provided data on the culture of species although these were not always produced on a commercial scale and the actual numbers of each species cultured were omitted as they were commercially sensitive. Culture initiatives by these organisations focus on the most popular species (i.e. kept by a high percentage of the surveyed hobbyists) with high numbers of clown fish and damsels, angel fish, dotty backs, blennies, gobies, cardinal fish and sea horses. Some families e.g. assessors and bettas, and dragonets and mandarins kept by only a small number of respondents are still produced by at least three organisations. Popular families from the hobbyist survey but with no industry culture include: tangs; butterfly fish; puffer and box fish; wrasse and trigger fish.

There are no records of teleost fish successfully reproducing asexually. However, a recent report [26] of automictic parthenogenesis by a white-spotted bamboo shark (*Chiloscyllium plagiosum*) producing parthenogens that survived for 5 years would indicate that this method may be viable for elasmobranchs. Invertebrates exhibit a much wider range of reproductive methods than fish. There is a hobbyist and commercial ‘trade’ in coral frags (small fragments of corals removed from the parent colony) with many species capable of being reproduced in this way. Asexual reproduction of some anemones is also possible [20], [21] and for fan worms, Murray et al., [16] have used the process of regeneration. Published culture methods exist for gastropods such as *Trochus niloticus* and *Turbo marmoratus* [29], [30] and crabs in the genus *Mithraculus* [31], [32], [33] although there are no other reports for other crab species collected for the trade. Alternative sources of gastropods have also recently been highlighted with the successful testing of a temperate species of Trochid top shell as an alternative to the tropical cleanup gastropods [34].

### Gap analysis

Generally the higher the quantity of a species imported, the greater the number of hobbyists keeping that organism; these provide a good indication of stock demand (Table 2, 3). The exception is the Serranidae family; a large family of fish that includes species from two groups of the online survey thus explaining the discrepancy. Only a small number of groups/families kept by the surveyed hobbyists were identified as amber or red in the traffic light system for both fish and invertebrates. The tangs, wrasse, grammas, starfish, anemones and hermit crabs were classed as red; groups with high demand and a low potential for this demand to be met by aquaculture. Trigger fish, butterfly fish, puffer and box fish and fan worms were categorised as amber with a medium stock demand with a low likelihood of this demand being met by aquaculture. The remaining groups/families were considered as green category species with low demand species such as lion and hawk fish and those with medium/high demand but high aquaculture potential such as blennies, gobies, clownfish and clams.

### Purchasing drivers

**Figure 1:**
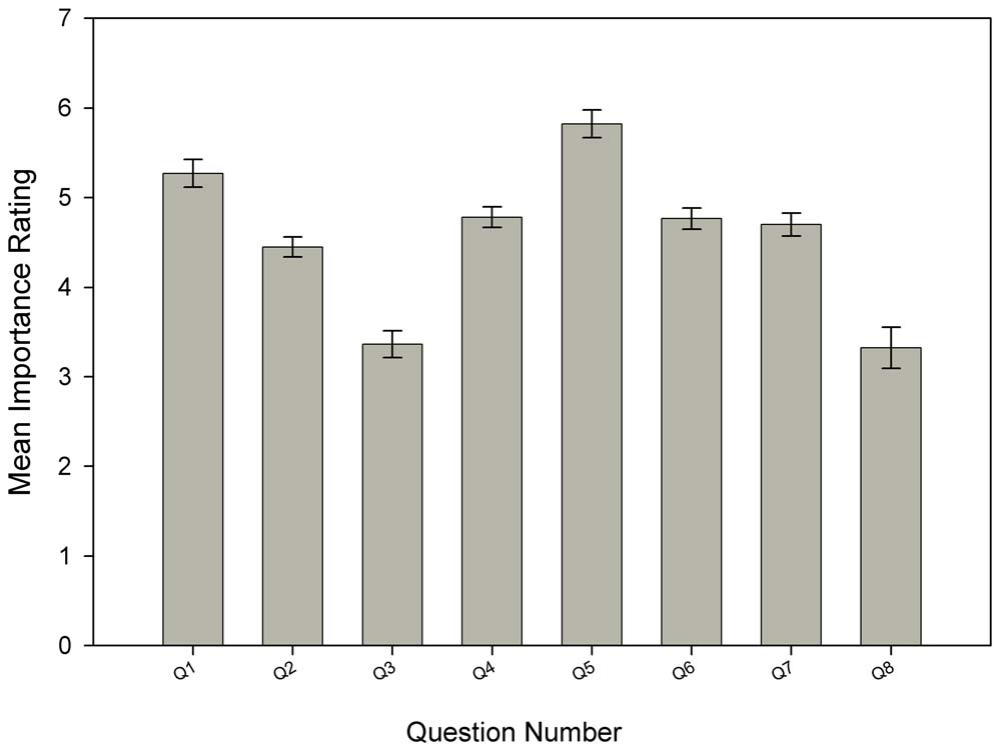
Summary of the most important factors when buying a new aquarium species. Summary of hobbyist responses when asked what the most important factors are when buying a new aquarium. The higher the mean score the greater the perceived importance. 1. It looks good; 2. Price; 3. My local shop recommended it; 4. It's easy to care for; 5. Compatibility; 6. It provides a function within the aquarium; 7. Collection source (tank bred or wild caught); 8. Other.

There were significant differences (K-W, H_7_ = 210, P = <0.001) in the mean (±SEM) scores of the ranked drivers affecting the purchasing behaviour of the hobbyist (Figure 1). The most important factors (i.e. highest mean scores in non-parametric post-hoc multiple comparison tests) are ‘compatibility with current stock’ and aesthetics (‘it looks good’). The second group: ‘collection source’; ‘it is easy to care for’; ‘it provides a function in the aquarium’; and ‘price’ are significantly lower than the most important factors. ‘My local shop recommends it’ and ‘other responses’ were of the least importance.

When asked if they would preferentially purchase a cultured or tank bred animal over one which was wild caught, only 3% of the respondents said “no”. Seventy six percent of the remaining respondents said they would preferentially buy cultured stock but 21% said it would depend on price. When asked what price premium they would be willing to pay the most popular response (30%) was a premium of 20%, but with 18% willing to pay a 50% premium.

### Information sources and availability

Ninety seven percent of responding hobbyists stated they would like to be offered more information on their organism at the point of sale. Information on whether the animal was cultured or wild-caught was the most popular response (91%). Of least importance was the provision of accurate scientific names (56%). When hobbyists were asked about their knowledge of current trade associations, only 55% had heard of the Marine Aquarium Council (MAC) and 61% Ornamental Aquatic Trade Association (OATA), but 62% of respondents claimed these had not been influential in the setting up of their aquarium.

### Benefits of the trade and the future

When questions were asked to understand the hobbyist's perceptions of the trade, the majority of respondents (69%) felt the trade increases understanding of coral reef ecosystems and 63% thought it informed aquaculture and breeding projects. Only 40% considered the aquarium trade encourages better protection of reefs but over half (51%) thought it was useful in providing jobs and money for those communities in developing countries. When asked about the future of the trade, an overwhelming majority (88%) thought that improvements in aquarium technology and equipment will continue and 92% wanted to see more cultured organisms available in the future. Seventy nine percent thought that hobbyists themselves would be responsible for expanding the range of animals they culture while 63% thought that the loss of coral reefs will reduce the number of animals available.

## Discussion

Best estimates indicate that there are at least 2 million global participants of the aquarium trade [2] but there have been no recent studies aimed at understanding the purchasing preferences and ideals of an aquarium hobbyist. Over 10 years ago Alencastro [35] undertook an online study of the hobby but our study is the first to provide an up-to-date assessment of the aquarium hobbyists' characteristics and motivations. A weakness of all online surveys is that not everyone has equal access to the internet [36] and it is likely that the respondents surveyed here were the most informed and committed. Despite the survey being published on global online forums, the majority of respondents were experienced hobbyists based in the UK. For the purposes of determining the biodiversity in the hobbyist tanks however, we believe that this demographic is likely to keep both the most popular and rare species; therefore providing a valuable contribution to a currently data-poor understanding of what is actually in the hobbyist tank compared to import data. The general biodiversity choices made by the responding portion of the hobby are also corroborated by patterns in the trade data presented by Rhyne et al [9] from Florida suggesting that despite the limitations of the survey method employed, the results are consistent with the wider trade. Furthermore, previous attempts to reach more broadly across the spectrum of industry stakeholders using different survey methods proved futile. For example, in a mail shot of 435 aquarium retailers across the UK Murray [37] achieved a zero response rate. Although the data generated for this study were from a focused group of respondents, it does provide a significant first step in gathering biodiversity data from the final consumer to better our understanding of the whole chain of custody in the marine aquarium trade.

### What do hobbyists keep?

The move in recent years to mini-reef ecosystems is supported by the survey results as only 8% of the respondents keep a fish-only system. Aquaria of 30–100 litres capacity (or even less) are now common and made possible by rapid technological advances especially in LED lighting. Many of the most popular species kept by the respondents (e.g. blennies and gobies) are small species and as reef-based tanks require key invertebrates, the popularity of corals and anemones is not surprising. The popularity of ornamental shrimp has been documented by Calado et al. [38], and our data support the role of shrimp as ‘feature invertebrates’ within the aquarium. Species termed ‘clean-up crew’ are those that control growth of unsightly and nuisance algal biofilms such as snails and hermit crabs and as a result, these groups are highly popular in the tanks of the respondents. Some starfish are also known to fulfil this role but their popularity is likely to be due to aesthetic appeal of some species e.g. *Fromia* spp. and *Linkia* spp.

Hobbyist literature stresses the importance of choosing compatible stock so it is reassuring to see that this is a key factor determining the purchases respondents make. With aesthetic appeal being the second most important factor, the balance between compatibility and aesthetic value in the aquarium is an ongoing issue that shows little sign of diminishing. Price was of surprisingly low importance in determining the choice of organism supporting Alencastro's [35] data that price does not constrain purchase decisions. The low importance in using advice from their local shop may reflect two things: firstly, the use of online information sources for advice and secondly, that the purchase of organisms from internet suppliers has become increasingly dominant. There are no data available (even from trade associations (K. Davenport, OATA, Pers. Comm.) on either of these potential reasons but revenue in the aquarium trade is likely to have moved away from traditional retail outlets towards online sources. This may also be a result of the experienced demographic surveyed and local advice may be of higher importance to those new to the hobby.

The reef aquarium is a facsimile; aiming to replicate the invertebrate and fish diversity found on a reef as closely as possible however, the vast majority of hobbyists only keep a small number of species found on a reef. Rhyne et al. [25] for example reported that 1802 species were imported into the US in 2005, but only 477 of these were imported in numbers over 1000 individuals. There will always be those hobbyists who want to keep rare/challenging species but most opt for well-known species, presumably to maintain compatibility and ensure aquaria success. Popular species will have the supporting information about their biology and care which is required to make an informed purchase decision. Without accessible species' information, the ‘risk’ of purchasing an incompatible organism may be too great although the underlying reasons for this narrow species choice requires further research.

Greater availability of cultured organisms was an overwhelming desire of hobbyists who responded to the survey, something that has not changed in 10 years since [35]. For the majority of hobbyists this is not dependent on price, and even for those who did say price was important, 65% were willing to pay premiums of up to, and including 20% extra. Two key issues should be considered with the results of the question. First is the saying “yes” phenomena when respondents agree to a statement that they feel it is what is socially acceptable and secondly, is the hobbyists' willingness to pay (WTP) for more sustainable aquarium products as it has been found that hypothetical WTP is often much higher than actual behaviour [39].

### Sources of cultured organisms

Aquaculture is increasingly cited as a priority solution to reduce pressures on coral reefs, as well as having important economic benefits for the trade [14], [16], [17], [18], [38]. The MBI data show that a significant number of species (the vast majority being fish) are reported as ‘successfully’ bred (Table 2). For example, 65% of the species of Pseudochromidae imported to the US in 2005 have been bred in captivity, a testament to the skill of hobbyists and the industry. However, successful breeding can mean anything from ‘inducing spawning’ to ‘keeping the larvae alive for 60 days post-larval settlement’ and so most successes do not lead to marketable-sized organisms; the true measure of successful aquaculture. Many species still face significant technological bottlenecks [17] and the vast majority of hobbyists involved in culture initiatives cannot progress their efforts due to technological and system limitations. The supply of cultured fish to the aquarium trade is therefore dominated by commercial organisations. The data provided by both the European and US organisations generally mirror the MBI pattern with around 10 families being the focus of their efforts and with significant success for some of those families (e.g. one has records for 25 species of Blennidae). The organisations have also focused on the same families with similar levels of success (e.g. eight of the nine organisations have been successful with clownfish).

Unlike fish, numbers of invertebrate reproduction records (Table 3) on the MBI are very low. Little is known about the reproductive habits of many invertebrates and as the MBI only accepts reports using sexual reproduction, many corals, some anemones and fan worms are excluded. Commercial organisations supplying the trade with invertebrates have also limited themselves to shrimp, snails, nudibranchs and Tridacninae clams.

### Assessing hobbyist biodiversity and current culture effort

Data on species cultured by the contributing commercial companies confirms that they have made considerable advances in their capacity to culture some organisms. A number of high demand groups whose wild stocks could be at risk if poorly managed are now nearly exclusively provided through aquaculture. Many of these have specific life-histories e.g. dermersal-spawning and male brooding which makes their culture relatively easy to achieve [17], [18]. Others such as corals are almost entirely supplied through asexual reproduction. For many of these popular species risk to wild stock is deemed low as demand can continue to be met by aquaculture. Even if a new species became very popular in the trade (as happened for the freshwater Red lined-torpedo barb, *Puntius* spp. [40]) it should be relatively easy to transfer understanding of reproduction in similar species to meet an increase in demand.

There is still however a substantial mismatch, high demand and a low or even non-existent aquaculture supply for a number of other groups including Tangs and wrasse. These two groups were flagged in this study as significant gaps in aquaculture effort (red category), meaning that they are in high demand based on hobbyist and trade data but there are no current culture initiatives to support their wild collection. Demand for wrasse was not separated from the other families but anecdotal observations of availability in the UK would indicate that they are also popular (Pers. Obs. G. Watson). Two species of wrasse (*Labroides dimidiatus* and *Pseudocheilinus hexataenia*) were in the top 20 species imported into the US [25] but despite their popularity there are no reports of breeding (commercial or otherwise) for any of these species.

Of the invertebrates, starfish, anemones and hermit crabs have been identified as gaps with high hobbyist demand and limited culture effort. There are scattered reports of asexual reproduction in anemones [27], [28] and a limited understanding of the reproductive systems of some heavily traded species [41], [42], [43], but these techniques do not seem to have been taken forward by the commercial organisations. This lack of knowledge transfer from the scientific literature to the industry is also apparent for *Mithraculus* crabs. No other information exists for any hermit crabs or starfish routinely traded [18]. We recommend that these red category groups are therefore priority candidates for wild stock assessment at a regional scale to ensure that local reefs can sustain current and future collection rates. It may be that some of these groups are collected in low numbers from numerous locations and are therefore of low concern, however, their high demand warrants further assessment to establish what a sustainable collection level is for each of these groups within a local site-specific coral reef system and alongside other human mediated pressures.

### Aquaculture and wild collection: an integrated approach

Unlike the freshwater ornamental trade where around 90% species are commercially bred; only 1–10% of marines are currently cultured [2]. Of those successful operations, most have been developed in countries such as Florida and Singapore where the constraints of poor infrastructure and economic instability are less prevalent [44], [14]. It is widely accepted however that aquaculture efforts would be most valuable in source countries where local fishers are reliant on the trade and where the benefits of integrating conservation and sustainability of local reef resources would be greatest [2], [45], [46]. This conflict in achieving successful marine ornamental aquaculture whilst safeguarding local fishers' income from wild collection and incentivising coral reef health must be considered in an ecosystem based approach.

Murray et al. [16], suggested that future aquaculture initiatives should be focused on developing simple and cheap culture technologies which can easily be transferred to local communities in developing countries. The authors present the use of regeneration as a method to culture fan worms and describe it operating in a small-scale community led project where fishers could propagate fan worms *in-situ* alongside other activities such as coral or live rock farming. Development of simple culture technologies in source countries alongside wild collection, and which could possibly contribute to coral reef rebuilding projects (e.g. [47]) should be encouraged. However, there is substantial evidence that many types of aquaculture have significant environmental impacts that do not fit with the concept of sustainability (see references cited within [48]) and therefore aquaculture will not be appropriate, possible or required for all species. The results of the current study show that 84% of fish and 68% of invertebrate species are green in the traffic light system and are either successfully cultured or have a limited demand suggesting they are low concern groups to ornamental fisheries managers at present. The remaining groups (assessed as amber or red traffic lights) generally have high demand coupled with a low potential for future aquaculture. We recommend that these are priority groups for fisheries assessors to evaluate if local collection of these groups is taking place within sustainable limits for that specific reef system.

### The concept of sustainability

The emergence of ‘green’ values among consumers, notably the sea fish food industry is now common (e.g. [48], [49], [50]) and proliferation of certified fish stocks confirms that some consumers will pay premiums associated with sustainably-sourced species or elevated welfare [46], [51]. Consumers must care about the sustainable practices before they will preferentially buy the product [52], but this purchasing choice relies on the consumer having an awareness of the origin and collection methods of the stock. The results of our survey indicate that this awareness may be lacking in the hobby at present as an overwhelming number of respondents (97%) wish to be offered more information at the point of purchase. In light of this, it is likely that it is a considerable challenge for the aquarium hobbyist to know what is or isn't from a sustainable source, whether this is collected from the wild or cultured. Additionally, only 40% of surveyed hobbyist felt that the trade encourages better protection of coral reefs and just over half (51%) that it provides socio-economic benefits to local fishers in source countries; a particularly interesting result given the widely held view among industry stakeholders (exporters, importers and scientists) that the aquarium trade incentivises the maintenance of a healthy reef ecosystem [8], [9], [16]. The failure of a certification system set up by the Marine Aquarium Council (of which only half of our most conscientious surveyed respondents were aware of), as well as a number of recent company-specific systems, is likely to have caused confusion among hobbyists over the concept of sustainability in the aquarium trade. To align the thinking that the aquarium industry could provide economic stability and incentivise reef maintenance between industry, scientists and consumers, we believe there is a requirement for a more transparent understanding of ornamental fisheries on a local scale, the contributions aquaculture makes and the ecological implications of collecting high demand species. A robust evidence base, including data on species traded, cultured and acquired by the consumer, is essential to inform the sustainable management of ornamental fisheries.

### The future

What does the industry look like in the future and how can the concept of a ‘sustainable’ aquarium industry be aligned from exporters to hobbyists? Some have highlighted the need for sustainable products using some form of labelling system [18], [45], [53]. The traceability and fidelity issues are considerable [54] and these methods will not be successful without substantial consumer education, business engagement, proactive leadership and substantial reform in exporting countries [55]. Management also requires reliable and extensive data which has already been shown to be extremely challenging to achieve [11]. Fujita et al. [56], have recently put forward methods for managing data-limited ornamental fisheries and linked to Community Based Management and Marine Protected Areas which could prove to be successful.

These approaches will take significant time and investment to implement especially those that will have to work across the numerous parts of a diverse industry. To focus the minds of all those involved external drivers could be applied. The implementation of legislation and regulations led by importing countries has already been suggested by Tissot et al. [55], with some recent examples. However, we believe that a certification scheme established with government support and underpinned by regulation is the most effective way to move the whole trade towards a self-regulating industry. Certification driven by government regulation would go some way to avoiding the issues surrounding the failure of MAC; prevent industry ‘greenwashing’ from the proliferation of multiple certification schemes; address issues of welfare, and ultimately, support the continued development of aquaculture alongside well managed fisheries, especially in exporting countries where the societal benefits would be the greatest. Costs for this type of approach would be substantial (a full economic analysis for all stakeholders is required), but an additional ‘tax’ on all live products might be an option as our survey has shown price is not an important factor in determining choice of organism.

## Supporting Information

Table S1 **Demographic and survey questions asked of responding hobbyists.** Multiple-choice restricted and text-box questions asked of the hobbyist (%, n = 314) to ascertain basic demographic information of the responding hobbyists and to assess their personal reasons for: keeping an aquarium and the geographical region/species on which it is based; the most important factors are when buying a new aquarium animal using a rating scale; preferences for a “sustainable” aquarium trade and the perceived view of the future of the industry.
(DOCX)
