# Supplementary material for: A Critical Assessment of Marine Aquarist Biodiversity Data and Commercial Aquaculture: Identifying Gaps in Culture Initiatives to Inform Local Fisheries Managers
Source: PLoS One. 2014 Sep 10;9(9):e105982. doi: 10.1371/journal.pone.0105982 (PMC4160189; doi:10.1371/journal.pone.0105982)
Supplement: Table S1 — Demographic and survey questions asked of responding hobbyists. Multiple-choice restricted and text-box questions asked of the hobbyist (%, n = 314) to ascertain basic demographic information of the responding hobbyists and to assess their personal reasons for: keeping an aquarium and the geographical region/species on which it is based; the most important factors are when buying a new aquarium animal using a rating scale; preferences for a “sustainable” aquarium trade and the perceived view of the future of the industry. (DOCX) [file pone.0105982.s001.docx]

.

|  | **Are you a...** | Hobbyist  Retailer  Wholesaler |
| --- | --- | --- |
|  | **What country do you live in?** | O Open question |
|  | **Male or female** | Male  Female |
|  | **Age group** | Under 20  21-40  40-60  60+ |
|  | **Where did you find this questionnaire?** | Advert in hobbyist magazine  Online hobbyist site  Online Forum  Other |
|  | **Why did you decide to set up your own marine aquarium?** | 1. Commercial- You work within the trade 2. Recommendation from friends/family 3. Scuba diving interest 4. Aesthetically pleasing in the home 5. Other |
|  | **Is your tank:** | 1. Reef-based 2. Fish only |
|  | **Is your tank based on a particular region? If yes, which region?** | 1. Yes 2. No |
|  | **Your tank: which animals do you own?** | List of fish and invertebrates (Table 3a, b for full lists) |
|  | **What's most important when buying a new animal for your tank? (Rank each answer with the most important being 1 and the least important 8. Only one rank per factor)** | 1. It looks good; 2. Price; 3. My local shop recommended it; 4. It's easy to care for; 5. Compatibility; 6. It provides a function within the aquarium; 7. Collection source (tank bred or wild caught); 8. Other. |
|  | **If given the choice, would you buy a cultured fish or invertebrate for your tank over a wild caught animal?** | 1. Yes 2. No 3. Depends on price |
|  | **If dependent on price, what premium would you be willing to pay?** | 1. 5% 2. 10% 3. 20% 4. 50% |
|  | **Of the statements listed below, tick which ones you agree with;** | 1. The marine aquarium trade provides jobs and money to communities in developing nations 2. The trade increases understanding of reef animals 3. The trade helps develop breeding projects and inform aquaculture |
|  | **Would you be interested in more information about your animal at the point of sale?** | 1. Yes 2. No |
|  | **If yes, what sort of information would interest you?** | 1. Origin country 2. When it was collected 3. If it is wild caught or cultured/tank bred 4. Scientific species names 5. Information on their biology and ecology in the wild |
|  | **Have you heard of; Marine Aquarium Council (MAC) and Ornamental Aquatic Trade Association (OATA)** | 1. Yes 2. No |
|  | **If yes, were they influential in choosing the stock in your tank?** | 1. Yes 2. No |
|  | **Do you have future plans for your tank?** | 1. Adding to, and developing your current tank 2. Setting up a bigger tank to replace your current one 3. Setting up an additional tank 4. Other (please specify) |
|  | **Which of the following statements about the future of the marine aquarium trade do you agree with?** | 1. Improvements in tank technology and equipment will continue 2. A wider range of animals will be available to purchase in the future 3. Reef conservation will lead to a reduction in the range of animals available 4. Loss of coral reefs will lead to a reduction in animals available to buy 5. More animals will be cultured/tank bred in the future 6. You would like to see more cultured animals available 7. Hobbyists will expand the range of animals they culture between themselves |
